# A Pictorial Review on Mastitis: Clinical Aspects, Imaging Features and Complications

**DOI:** 10.3390/jimaging12050181

**Published:** 2026-04-23

**Authors:** Giovanna Romanucci, Claudia Rossati, Marco Conti, Delia Moretti, Gianluca Russo, Francesca Fornasa, Carlotta Rucci, Oscar Tommasini, Paolo Belli, Rossella Rella

**Affiliations:** 1 UOSD Breast Unit ULSS9, Ospedale di Marzana, Piazzale Lambranzi, 1, 37142 Verona, Italy; claudia.rossati@aulss9.veneto.it (C.R.); francesca.fornasa@aulss9.veneto.it (F.F.); 2 UOC Diagnostica per Immagini, Ospedale G.B. Grassi, Via Gian Carlo Passeroni, 28, 00122 Rome, Italy; marco.conti@aslroma3.it (M.C.); oscar.tommasini@aslroma3.it (O.T.); rossella.rella@aslroma3.it (R.R.); 3 Facoltà di Medicina e Chirurgia, Università Cattolica Sacro Cuore, Largo F. Vito 1, 00168 Rome, Italy; delia995moretti@gmail.com (D.M.); gianluca7russo@gmail.com (G.R.); paolo.belli@policlinicogemelli.it (P.B.); 4 UOC di Radiologia Toracica e Cardiovascolare, Dipartimento di Diagnostica per Immagini, Radioterapia Oncologica ed Ematologia, Fondazione Policlinico Universitario Agostino Gemelli IRCCS, Largo A. Gemelli 8, 00168 Rome, Italy

**Keywords:** mastitis, breast infections, breast abscess, breast cancer mimickers, lactational mastitis, non-lactational mastitis, mastitis treatment, mastitis imaging

## Abstract

Breast mastitis is a common condition that can be found during clinical practice, challenging the clinician, who must reach the correct diagnosis among the many differentials, to properly treat the underlying pathology. In this review, we aim to provide clinicians and radiologists with an overview of the various forms of mastitis, focusing on clinical presentation, etiological subtypes, imaging appearances across modalities (e.g., ultrasound, mammography/tomosynthesis, contrast enhanced techniques, MRI), related complications, and the typical imaging takeaways. Our goal is also to provide tools for the correct differential diagnosis between various forms of mastitis, breast cancer and other inflammatory breast pathologies. A computerized literature search using PubMed and Google Scholar was performed by authors, entering various keywords (e.g., “mastitis”, “breast infections”, “breast abscess”, “breast cancer mimickers”, “lactational mastitis”, “non lactational mastitis”, “mastitis imaging”, “rare forms of mastitis”). Articles published between 2002 and 2025 were taken into consideration. The authors selected various eligible studies, scientific articles and extracted data to cover the whole spectrum of mastitis clinical presentation and underlying pathology. Authors divided the mastitis spectrum into “lactational” and “non-lactational” forms. Between the second group, periductal mastitis, idiopathic granulomatous mastitis, and rarer forms are taken into consideration. Our review has several limitations: it is a narrative and not systematic review and has limited generalizability of rare subtypes because of the case report driven evidence, heterogeneity of selected studies and potential selection bias. It supplies imaging from various clinical cases, which can be useful to familiarize with the pathology spectrum. In conclusion, breast mastitis is a challenge for breast radiologists and clinicians, familiarity with this condition is crucial to make a correct differential diagnosis. Further studies are needed on rarer subtypes.

## 1. Introduction

**Figure 1:**
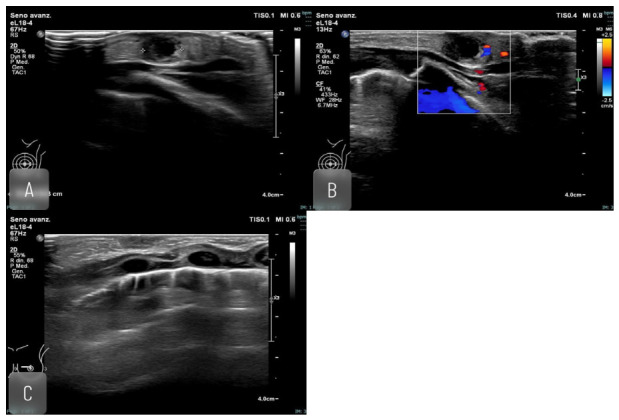
One month old newborn, with swollen left breast. US reveals pericentimetric hypoechoic breast lesion compatible with abscess. Inflammatory axillary lymph node was detected. Antibiotic therapy was administered with full recovery both clinical and ultrasound. (**A**,**B**) US imaging, breast lesion before medical therapy; (**C**) US imaging, breast after medical therapy.

Breast mastitis is a relatively common inflammatory condition that can occur at any stage of life (Figure 1) [1] but is prevalent in women during the breastfeeding period. It is defined as inflammation of the breast with or without infection. Infectious mastitis may be classified as lactational (puerperal) or non-lactational. 

Infectious forms occur more frequently in breastfeeding women (LM/PM—lactational or puerperal mastitis). Contributing factors include the prevalence of infectious diseases, limited patient education, and inadequate breastfeeding techniques. Non-lactational infectious mastitis (NLM) can result from various pathogens. Staphylococcus is the most frequent, but up to 30% of cases are polymicrobial (e.g., Enterobacteriaceae, Peptostreptococcus, Propionibacterium, Bacteroides). Rare causes include *Mycobacterium tuberculosis*, non-tuberculous mycobacteria (NTM), and *Corynebacterium* spp., which may mimic idiopathic granulomatous mastitis (IGM) [2]. Among non-lactational mastitis, the two major entities are periductal mastitis and idiopathic granulomatous mastitis, both of which are chronic inflammatory diseases that affect primarily young women [3,4]. Clinically, non-lactational mastitis ranges from acute infection to chronic disease, granulomatous, or fibrosing processes, and often poses diagnostic challenges due to overlap with both benign and malignant breast conditions.

Non-lactational forms present significant diagnostic challenges in imaging: while conventional techniques such as ultrasound remain the first-line imaging modality, advanced techniques such as MRI are increasingly valued for their role in complex or atypical cases [5]. Effective management requires a multidisciplinary approach integrating clinical, imaging, and, when necessary, histopathological findings to prevent misdiagnosis and overtreatment. This review aims to provide an up-to-date overview of the diverse causes of mastitis, identify various histopathological subtypes, and highlight the key clinical, radiological features and advances in imaging technologies.

## 2. Epidemiology

Breast infections are the most common benign breast disease during pregnancy and the puerperium. Lactational mastitis is common, the global prevalence of mastitis in lactating women ranges from 1% to 10%, affecting approximately one in four breastfeeding women during the first 26 weeks postpartum, and decreases gradually thereafter [4].

Non-lactational mastitis (NLM) refers to a heterogeneous group of inflammatory breast disorders unrelated to breastfeeding. It includes central periareolar infections, often periductal mastitis (PDM) or duct ectasia. This entity is typically linked to cigarette smoking and chronic inflammation of the central lactiferous ducts, while peripheral abscesses, involving the outer portions of the breast are less frequently associated with smoking or duct ectasia and may result from primary infections, local trauma, or systemic conditions such as diabetes, idiopathic granulomatous mastitis (IGM), or a rare chronic inflammatory disorder histologically characterized by non-caseating lobular granulomas. While often idiopathic, IGM may be associated with autoimmune phenomena, infectious agents [6] (e.g., *Corynebacterium kroppenstedtii*), or hormonal factors [7]. The most common non-lactational mastitis are periductal mastitis (PDM) observed in 5–9% of young non-lactating women and idiopathic granulomatous mastitis (IGM), mainly in women within 5 years of childbirth [7,8,9].

Another rare form of non-lactational mastitis is represented by tuberculous mastitis, which remains uncommon even in tuberculosis (TB)-endemic countries, with a reported incidence of 0.1–3% [10].

Among the complications of both lactational and non-lactational mastitis is the development of a breast abscess that occurs in approximately 0.4–3% of women with mastitis, with most cohort studies reporting around 0.4–0.5% incidence among breastfeeding women.

Breast abscesses in lactating and non-lactating women are two distinct clinical entities, each with specific pathogenesis: lactational abscesses arise from inadequate drainage during breastfeeding and are most often caused by Staphylococcus aureus and Streptococci; MRSA is increasingly seen. Non-lactational abscesses are more frequently observed in women toward the end of their reproductive years. They occur outside lactation, 90% often sub-areolar and linked to smoking, diabetes, ductal pathology; caused by mixed microbial flora; have a higher tendency to recur, and warrant malignancy work-up [7,11].

## 3. Classification and Pathogenesis

### 3.1. Lactational Mastitis

Lactational mastitis is an inflammation of the breast that is rare during pregnancy but common in the postpartum period, often influencing the decision to discontinue breastfeeding [4], with 1 in 4 women citing mastitis as the determining factor. The underlying causes of this condition are multifactorial: during breastfeeding and when the epithelial barrier is compromised, the infant can transmit microorganisms to the nipple and areolar region. Milk stasis represents a significant predisposing factor (resulting from factors such as nipple injury, latch difficulties, oversupply, skipped feedings, or the use of nipple shields) as breast milk provides a favorable environment for bacterial proliferation [12]. It manifests with systemic symptoms such as tenderness, swelling, redness, and increased temperature in the affected breast and local symptoms such as focal, firm, erythematous, swollen, and painful areas of one breast [13].

One potential contributing factor is the disruption of the breast microbiome, known as mammary dysbiosis, which may involve a bacterial imbalance rather than a straightforward infection. A disrupted milk microbiome—affected by factors such as antibiotic use, probiotic supplementation, hyperlactation, ductal narrowing, and the mechanical stress of breastfeeding—can contribute to this inflammatory condition. The source of infection is usually *Staphylococcus aureus* or a *Streptococcus* species from the infant’s nasal and oral cavities; however, these bacteria are also found in the healthy milk microbiome, making their role more complex [14].

### 3.2. Non-Lactational Mastitis

#### 3.2.1. Periductal Mastitis (PDM)

**Figure 2:**
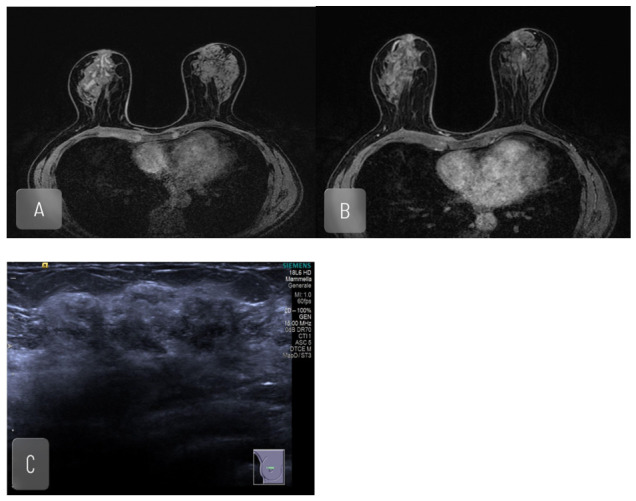
Sixty yo. woman with known left breast carcinoma. A staging MRI was performed, which confirmed left breast carcinoma and revealed a non-mass-enhancement area, extending posteriorly from the nipple. A core needle biopsy was performed, which revealed diffuse dilation, including cystic, of the ducts containing pulpy material. No suspicious macroscopic and microscopic lesions were observed. Fibrocystic mastopathy with massive chronic periductal lympho-histiocytic inflammation (e.g., periductal mastitis) was diagnosed. (**A**) MRI, basal T1 imaging; (**B**) MRI, contrast enhanced imaging revealed large non-mass enhancement area; (**C**) US imaging, revealed a large hypoechogenic area.

PDM, also known as plasma cell mastitis or periareolar mastitis, is a benign, chronic inflammatory disease with high risk of recurrence after treatment (Figure 2). It often develops in non-lactating women and accounts for 1–2% of all breast diseases. It typically involves the major ducts of the breast of the periareolar region [15]. Main clinical manifestations include non-cyclical mastalgia, nipple retraction, nipple discharge, subareolar breast lumps, and, in some cases, periareolar abscesses or breast fistulas and evolve in accordance with the stage and progression of the underlying inflammatory process: acute forms mimic suppurative mastitis with erythema, swelling, pain, abscesses, and systemic symptoms (fever, malaise); subacute forms show reduced systemic symptoms, but local inflammation persists with a palpable lump and dark red areolar skin. Chronic forms involve deep-seated areolar masses, recurrent flare-ups, and possible draining sinus tracts. The pathogenesis and etiology of PDM remain unclear, though several possible mechanisms have been proposed, including hormonal influences, ductal obstruction, smoking, and bacterial infection. Obstruction of lactiferous ducts may lead to their dilation and rupture due to retained secretions, allowing bacterial entry, which triggers inflammation that can extend to surrounding tissues and result in abscesses or fistulas [15].

Smoking is a significant risk factor for PDM due to its harmful effects on epithelial cells and ductal tissue, which promote bacterial colonization and anaerobic growth [16].

Conditions like obesity, nipple inversion, and hormonal disturbances, including hyperprolactinemia, have also been cited in studies as contributing to duct dysfunction and inflammation. However, these associations are primarily based on small case studies, lacking robust evidence. Bacterial involvement in PDM remains controversial. Although some studies identify common pathogens like Staphylococcus aureus and anaerobic organisms, others have reported sterile lesions, suggesting that immune responses following bacterial infiltration may play a more prominent role than the infection itself [9].

Although rare, PDM is painful, has a prolonged course, and shows a high risk of recurrence. No standardized treatment exists; conservative therapy with antibiotics alone has shown poor results. Smoking cessation is recommended, as smoking is a potential risk factor. Surgery is currently the most adopted treatment approach [17].

#### 3.2.2. Idiopathic Granulomatous Mastitis (IGM)

**Figure 3:**
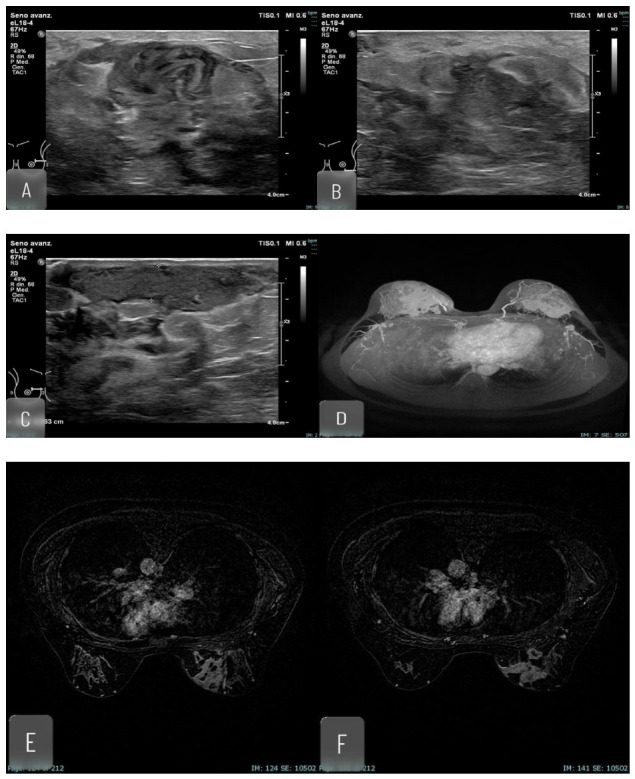

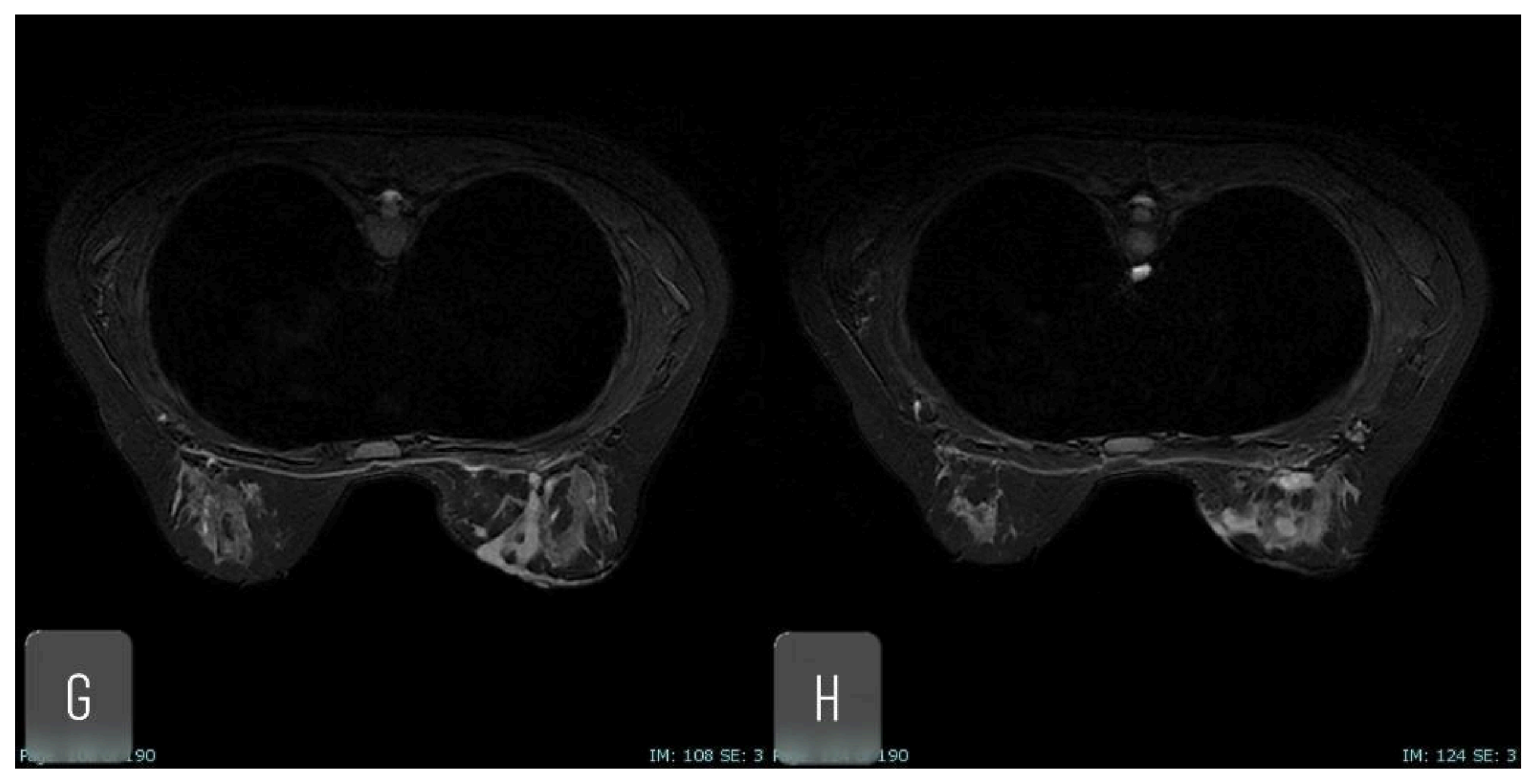
Thirty-five yo. woman, 2 weeks postpartum, with clinical picture compatible with lactational mastitis. No significant abscesses detectable with ultrasound. Ultrasound detects tubular pattern. An MRI was performed, which detected multiple coalescent contrast enhanced areas; no abscesses were found. A biopsy was performed which detected chronic phlogistic lymphoplasmacellular infiltrate. (**A**–**C**) US imaging, no abscesses were found; tubular pattern is detectable; (**D**) MRI, MIP; (**E**,**F**) MRI, Contrast enhanced imaging; (**G**,**H**) MRI, T1 imaging.

IGM, also known as non-puerperal mastitis or granulomatous lobular mastitis, is a rare and perplexing breast disorder with a prevalence of 2.4 per 100,000 individuals that predominantly affects young women of reproductive age, often occurs postpartum or during lactation and is characterized by noncaseating granulomas within breast lobules (Figure 3). IGM is more frequently observed in developing countries, affects all ethnic groups but appears to be more common among individuals of Asian, Hispanic, and Middle Eastern origin, although no ethnicity has been definitively shown to have a significantly higher risk.

IGM typically appears within 5 years postpartum and is associated with breastfeeding, with an average onset age of 33–38 years [8]. Although the etiology of IGM remains unclear, it has been suggested to be associated with autoimmune processes. 

Clinically, IGM typically presents unilaterally, although bilateral cases have been reported. Common symptoms include pain, inflammation, erythema, nipple discharge, and skin changes such as ulceration or sinus tract formation. A palpable mass—the most frequent finding—can occur in any breast quadrant, except for the subareolar region.

A grading system was developed to correlate the severity of IGM with clinical features and breast mass size. Mild IGM is characterized by a breast mass <2 cm with no associated ulcers or fistulas and minimal pain. Moderate IGM involves a breast mass measuring between 2 and 5 cm, with fluid collections that may require aspiration drainage, one fistula, and a small amount of discharge. Severe IGM is identified by a breast mass of >5 cm, severe pain, the presence of multiple fistulas, and ulcers that discharge >20 mL daily. This system is instrumental in evaluating the extent of the disease and determining appropriate treatment strategies. 

Generally, granulomatous mastitis can be classified as specific or non-specific. In the pathophysiology of IGM, it is hypothesized that ductal epithelial damage leads to the leakage of secretions into the lobular stroma, triggering a local immune response with infiltration of inflammatory cells and granuloma formation. Although the exact cause is unknown—hence the term “idiopathic” in most cases—environmental triggers are thought to initiate the condition in genetically predisposed individuals. Three main hypotheses for its pathogenesis are: autoimmune mechanisms, infectious agents, and hormonal imbalances.

Specific granulomatous mastitis is associated with identifiable causes like infections or systemic autoimmune diseases. In some studies, non-specific granulomatous mastitis is subcategorized into IGM, where no clear etiology is determined, and cystic neutrophilic granulomatous mastitis, which is associated with Corynebacterium infection. 

Several factors have been implicated in the development of IGM, including pregnancy, lactation, hyperprolactinemia, trauma, diabetes, and hormonal contraceptive use. These factors may increase breast secretions or disrupt the immune microenvironment, contributing to local inflammation. Among these, pregnancy and lactation have been consistently linked to IGM, likely due to residual milk stasis that damages lobules and induces hypersensitivity or immune reactions. Corynebacterium species, particularly C. kroppenstedtii, are increasingly recognized as possible pathogens in IGM, although their precise role is debated, as they are part of the normal skin flora [18].

Other proposed mechanisms include autoimmune pathways, though direct evidence is limited. Autoimmune disorders (e.g., erythema nodosum or arthritis) sometimes coexist with IGM, supporting an immune-mediated component. Inflammation and loss of acinar structure can lead to microabscesses, which may coalesce to form larger abscesses [8,19,20].

#### 3.2.3. Less Common Forms

**- Diabetic Mastopathy:** diabetic mastopathy (DMP) is an uncommon, benign fibroinflammatory breast condition, predominantly affecting women with longstanding type 1 diabetes. Despite its non-malignant nature, it often clinically and radiologically mimics breast cancer (BC), making diagnosis particularly challenging. Patients typically present with painless, irregular, and palpable breast masses, which may be unilateral or bilateral and recurrent. Imaging findings—such as spiculated margins and heterogeneous echotexture on ultrasound—can closely resemble those of malignancy. Therefore, a multidisciplinary approach including clinical assessment, imaging, and biopsy is essential to establish the diagnosis and rule out cancer. Histologically, DMP is marked by sclerosing lymphocytic lobulitis, a finding also seen in some autoimmune diseases, which limits its specificity to diabetes. Due to its resemblance to malignancy, DMP is frequently over diagnosed, often leading to unnecessary surgical procedures. Although several reports exist, most are isolated case studies, and there remains limited data on the clinical behavior of DMP and its potential link to breast cancer, especially in immunocompromised or autoimmune patients [21].

**- Eosinophilic Mastitis:** Eosinophilic mastitis is a rare, benign inflammatory breast condition characterized by eosinophil-rich infiltration of the breast stroma. Though it can clinically mimic breast cancer, it may occur without systemic eosinophilia or allergic disease, making diagnosis challenging [22]. Most cases are linked to conditions like hypereosinophilic syndrome, asthma, or autoimmune disorders, but isolated cases are rare and may develop during lactation, showing spontaneous resolution without the need for treatment—emphasizing the variable clinical course of this entity. Given its potential for misdiagnosis, eosinophilic mastitis should be considered when histology reveals eosinophilic inflammation, after excluding infection and malignancy. Recognizing its distinct pathological features is key to avoiding unnecessary procedures [23].

**- Lupus Mastitis:** Lupus mastitis is a rare but clinically relevant manifestation of lupus, frequently misdiagnosed due to its resemblance to infectious or malignant breast lesions. Due to the higher incidence of autoimmune disorders in females, lupus mastitis predominantly affects premenopausal women, most commonly around the age of 40. Nevertheless, cases have been reported across a broader age range (18–70 years), with male involvement being exceedingly rare [24].

Based on case reports, a non-invasive diagnosis may be possible in patients with compatible clinical and radiological features. Given its rarity, the incidence of lupus mastitis (LM) is often extrapolated from data on lupus panniculitis, a form of subcutaneous fat inflammation that occurs in approximately 2–3% of patients with systemic lupus erythematosus (SLE). LM represents a localized subset of lupus panniculitis limited to breast tissue. To date, no definitive incidence rate for LM has been established [25].

The authors suggest that a diagnosis of lupus mastitis may support a diagnosis of SLE even when current classification criteria are not fully met.

Developing specific diagnostic guidelines may enhance clinical management and reduce the risks of misdiagnosis or overtreatment.

**- IgG4-Related Mastitis:** IgG4-related mastitis is a frequently overlooked form of chronic fibroinflammatory breast disease. Cases previously classified as non-specific mastitis or inflammatory pseudotumors may represent this entity when reassessed with appropriate immunohistochemical staining.

Since systemic IgG4-related disease (IgG4-RD) criteria may not fully capture its breast-specific features, dedicated diagnostic guidelines are needed. Proper identification can prevent unnecessary surgical interventions and enable tailored immunosuppressive therapy [26].

**- Tuberculous mastitis:** tuberculous mastitis (TM) is a rare form of extrapulmonary tuberculosis that primarily affects women of reproductive age, particularly during lactation or the postpartum period. It often mimics breast cancer due to overlapping symptoms such as firm lumps, fistulas, pain, nipple retraction, skin involvement, and axillary lymphadenopathy [10]. TM can occur as an isolated breast lesion or as part of systemic TB. Diagnosis is challenging and often relies on cytology or histopathology demonstrating granulomatous inflammation, as imaging findings are generally non-specific. First-line treatment is anti-tubercular therapy, while surgery may be needed in resistant or complicated cases.

## 4. Diagnostic and Imaging Features in Mastitis

### 4.1. Clinical Presentation and Supporting Laboratory Findings

Lactational mastitis is primarily diagnosed through clinical history and examination. Patients presenting with systemic symptoms (e.g., fever, tachycardia) persisting for more than 24 h should be assessed for mastitis. It should also be considered in those without systemic signs who fail to improve with supportive care. If there is no clinical response after 48 h of empirical antibiotic therapy, further evaluation is warranted. This may include milk cultures to detect resistant organisms (such as MRSA). In cases of severe infection with suspected bacteremia, blood cultures can be obtained, although they are not routinely indicated [12].

Non-lactational mastitis requires a more comprehensive approach, combining imaging, pathology, and microbiological testing to distinguish between periductal mastitis (PDM) and idiopathic granulomatous mastitis (IGM), while ruling out malignancy and other differentials. Laboratory markers like CBC and CRP (complete blood counts and C-reactive protein) may assist in identifying systemic infection or severe inflammation.

### 4.2. Imaging

**- Ultrasound:** Ultrasound (US) is the primary imaging modality for evaluating mastitis in patients of all ages. In lactational mastitis, ultrasound typically reveals a range of inflammatory findings. These may include diffuse or focal skin thickening, inhomogeneous breast parenchyma, and increased echogenicity of the subcutaneous fat. Hypoechoic linear striations are frequently seen within the subcutaneous tissue, consistent with edema and fluid accumulation. An irregular hypoechoic area or mass may be present, occasionally containing heterogeneous fluid, indicative of a developing abscess (different types of mastitis with imaging tools and imaging featured are listed in Table 1). On color Doppler imaging, these areas often demonstrate peripheral hyperemia, reflecting active inflammation. The sonographic features of periductal mastitis (PDM) typically include dilated intramammary ducts with internal echogenic filling defects and increased surrounding vascularity. Complications are similar to lactational mastitis and include abscess and fistula formation [27].

The sonographic appearance of IGM is variable, but the most common feature is an irregular hypoechoic mass, often with tubular extensions. These extensions, which tend to coalesce, create a reticular pattern representing the lobular spread of inflammation. Lesions typically appear parallel to the skin. Lesions in periductal mastitis and IGM may appear irregular and often overlapping, but ductal dilatation >3 mm in PDM with internal echoes or mobile secretions is more typical of PDM, but not exclusive. Color Doppler US often reveals hypervascularity within the lesion and surrounding tissues. In advanced disease, abscesses may form, with prevalence ranging from 6.6% to 54% in the literature. Additional sonographic signs may include: skin thickening, edema and increased echogenicity of subcutaneous fat, or reactive axillary lymphadenopathy with a thickened cortex but preserved fatty hilum.

Elastography is an innovative complementary imaging technology that enhances the diagnostic capabilities of B-mode US by evaluating tissue stiffness, it is effective in distinguishing benign from malignant breast lesions and can improve the specificity of traditional US when using the Breast Imaging Reporting and Data System (BIRADS) to differentiate IGM-related inflammatory breast changes from breast cancer; Ref. [28] evaluates tissue stiffness, which can help differentiate mastitis from cancerous lesions. Cancerous tissues tend to be stiffer than inflammatory tissues.

Infectious mastitis may progress to breast abscess formation, which can occur in association with lactation (puerperal abscess) or independently of pregnancy (nonpuerperal abscess). Puerperal abscesses are typically peripheral and clinically apparent, whereas nonpuerperal abscesses—more frequent in younger women—are usually periareolar, present greater diagnostic difficulty, and are associated with poorer prognosis and higher recurrence rates. Smoking and diabetes are recognized as risk factors for the nonpuerperal form. On mammography, mastitis and abscess may manifest as skin thickening, asymmetry, mass-like densities, or architectural distortion. Ultrasound typically reveals one or more hypoechoic fluid collections of variable morphology, often confluent and multiloculated, with a thick echogenic capsule and marked peripheral vascularity, features that may mimic malignancy.

**- Contrast-Enhanced Ultrasound (CEUS)**: CEUS is an advanced imaging modality used in the evaluation of mastitis. It involves the intravenous injection of microbubble-based contrast agents, such as sulfur hexafluoride or perflutren, which circulate rapidly and allow real-time assessment of tissue perfusion and microvascular architecture. Compared to conventional B-mode ultrasound, CEUS improves the characterization of inflammatory breast lesions [29]. Contrast-enhanced ultrasound (CEUS) is not routinely used or validated for the evaluation of lactational (puerperal) mastitis. The current literature does not support its application in this setting. While CEUS has been explored in non-puerperal subtypes such as granulomatous and plasma cell mastitis, no clinical studies or guidelines recommend its use in lactational mastitis. In mastitis, CEUS can reveal distinctive perfusion patterns—such as smooth-edged perfusion defects in plasma cell mastitis or diffuse enhancement in granulomatous mastitis—which aid in differential diagnosis. The technique is well tolerated, with a favorable safety profile and no significant adverse effects. A recent study compared the contrast-enhanced ultrasound (CEUS) features of GLM and breast cancer using both quantitative and qualitative methods. The results demonstrated that GLM showed significantly higher time-to-peak (TTP) values and lower wash-in slope (WIS) values on CEUS compared to breast cancer. These CEUS-derived quantitative parameters provided additional diagnostic value over conventional ultrasound, with TTP showing a sensitivity of 73.33% and a specificity of 84.48%, and WIS also demonstrating high sensitivity and specificity. This approach may help reduce unnecessary biopsies in patients with GLM by improving differentiation from breast malignancies [30]. Radiologist expertise is critical for accurate mastitis diagnosis. Experienced interpreters can distinguish inflammatory changes from malignancy or benign lesions, even in challenging cases such as granulomatous mastitis. Proficiency in advanced ultrasound techniques, including elastography and contrast-enhanced ultrasound, enhances diagnostic accuracy by leveraging quantitative parameters—such as time-to-peak (TTP) and wash-in slope (WIS)—to differentiate inflammatory from malignant processes [31].

**- Mammography:** Mammography plays a limited role in the initial evaluation of mastitis and breast abscesses due to its reduced diagnostic sensitivity in the acute phase and the potential discomfort caused by compression of an inflamed or abscessed breast. The radiographic features of infectious breast conditions are often non-specific and may include no visible abnormalities, architectural distortion, spiculated masses, skin thickening or retraction, microcalcifications, and areas of focal or diffuse increased density [14]. These findings can closely resemble those of malignancy, making differential diagnosis challenging. For this reason, mammography is best reserved for the post-acute phase, once inflammation has subsided, to allow for detection of any underlying lesions. It is particularly indicated in women over 40 years of age, for cases with atypical or persistent symptoms, or when there is clinical suspicion of malignancy.

In some cases, mammography may appear normal, especially in early stages. Characteristic thick, linear, cigar-shaped or rod-like calcifications are seen in certain types of mastitis, such as those associated with duct ectasia.

**- Digital Breast Tomosynthesis (DBT):** Although ultrasound remains the first-line imaging modality for acute mastitis, DBT has shown added value in selected cases. Studies suggest that DBT improves lesion detection and characterization compared to conventional mammography, particularly by identifying masses that are not visible on standard 2D imaging. It enhances the visualization of benign features—such as tubular extensions and low-density areas—thus reducing the risk of overestimating malignancy and aiding in more accurate BI-RADS categorization. However, the current literature does not support the routine use of DBT in the acute setting, though it may be useful in cases with persistent symptoms, indeterminate findings, or in women with dense breast tissue [32].

**- Contrast-Enhanced Mammography (CEM):** The current literature on contrast-enhanced mammography (CEM) focuses primarily on breast cancer diagnosis and staging, problem-solving for suspicious mammographic findings, high-risk screening, and preoperative or post-neoadjuvant therapy assessment [33].

Some studies note that benign inflammatory lesions, such as mastitis, may cause false positives due to increased vascularity, but no data support the use of CEM for mastitis diagnosis [34]. 

**- Magnetic Resonance Imaging (MRI)**: MRI is used in the evaluation of mastitis, particularly in non-puerperal cases or atypical presentations, when differential diagnosis with breast malignancy is needed. Therefore, although MRI is not useful as a first-line diagnostic tool not typically used as a first-line tool for mastitis, it can help differentiate mastitis from cancer, especially in complex cases or when other imaging modalities are inconclusive. MRI is used in the assessment of lactational mastitis only in selected cases. Primary indications include differentiating inflammatory processes from malignancy, evaluating the extent of disease, and surgical planning when a tumor is suspected [35].

Typical MRI findings in lactational mastitis include marked background parenchymal enhancement (BPE), often symmetric and bilateral, with a non-mass-like enhancement pattern showing regional or diffuse distribution, and associated edema [36]. In cases of abscess, a well-defined lesion with peripheral rim enhancement and a hyperintense T2-weighted center may be observed. MRI is considered safe during breastfeeding, as the use of gadolinium-based contrast agents is not contraindicated in lactating women.

The most frequent MRI findings in idiopathic granulomatous mastitis (IGM) include segmental heterogeneous enhancement and ring-like abscess formations—features consistently reported across multiple studies. In mass-like lesions, common characteristics are round shape, smooth margins, and rim enhancement, with peripheral rim enhancement being the most typical finding suggestive of abscess. Despite other benign features, such lesions are often classified as BI-RADS 4 due to diagnostic uncertainty.

Non-mass enhancement (NME) lesions show predominantly segmental or, less commonly, linear enhancement patterns [37], which may mimic ductal carcinoma in situ. Irregular masses, histologically attributed to non-caseating granulomas, are seen in a few cases and represent a feature with high predictive value for malignancy, often leading to misdiagnosis. Sinus tracts are rare. MRI is more sensitive than mammography or ultrasound in detecting and characterizing lesions in non-lactational mastitis, it provides better assessment of disease extent, but its findings remain non-specific and insufficient for differential diagnosis between IGM and breast carcinoma.

### 4.3. Microbiology and Pathology Investigations

A biopsy is not recommended to evaluate lactational mastitis, it is not routinely indicated and should be reserved for lesions that persist following appropriate treatment, for non-resolving palpable masses, or in the presence of suspicious imaging findings (BI-RADS category 4 or 5, and selected category 3 lesions).

It is indicated in cases of mastitis when there is clinical or radiological suspicion of malignancy, when atypical features are present, or in cases of failure of clinical resolution following antibiotic therapy [38].

Indications for biopsy include the detection of solid masses, irregular margins, persistent asymmetries, or lack of clinical resolution after antibiotic therapy.

Non-lactational mastitis, encompassing PDM and IGM, demonstrates distinct yet overlapping histopathological features. A biopsy is the gold standard for diagnosing non-lactational mastitis, with core needle biopsy showing higher sensitivity (96%) than fine-needle aspiration (21%). Given that the clinical features of IGM overlap with those of breast cancer a breast biopsy must be performed for differential diagnosis. PDM typically shows chronic inflammation with ductal dilatation and plasma cell infiltration, while IGM is defined by necrotizing granulomatous inflammation centered on lobules without caseation. Key histological findings in IGM include epithelioid histiocytes, multinucleated giant cells, and neutrophil-dominated microabscesses [9,15].

### 4.4. Complications and Oncologic Correlation in Mastitis

If inadequately managed, mastitis can progress to suppurative complications. Breast abscesses develop in approximately 3–11% of cases, with risk factors including advanced maternal age at first pregnancy (>30 years), prolonged gestation (>41 weeks), and prior mastitis episodes. Both periductal mastitis (PDM) and idiopathic granulomatous mastitis (IGM) can result in abscess or mammary fistula formation (1–2% incidence) and are prone to recurrence, potentially leading to fibrosis, permanent scarring, and breast deformity.

Clinically and radiologically, granulomatous mastitis may mimic inflammatory breast cancer. In puerperal mastitis unresponsive to appropriate antibiotics, malignancy should be actively excluded. In such cases—as well as in atypical or recurrent presentations—histopathological confirmation is essential. Diagnostic sampling methods include fine-needle aspiration, core-needle biopsy, or surgical excision, with pathology required to exclude carcinoma and identify infectious causes. Epidemiological studies suggest a significant association between non-lactational mastitis and subsequent breast cancer risk. In a Taiwanese population-based cohort (*n* = 3091; mean age 37.9 years), the adjusted hazard ratio (aHR) for breast cancer was 1.94 (95% CI: 1.30–2.90) compared to controls, with the highest risk in women <50 years, those with lower socioeconomic status, and hormonal therapy users. Risk increased with the number of mastitis episodes [39] A German retrospective cohort likewise found elevated breast cancer risk post-mastitis (HR 1.37; 95% CI: 1.11–1.70), highest in women >50 years (HR 1.73; 95% CI: 1.25–2.40).

## 5. Radiologic Challenges

### Overlap with Malignancy

**Figure 4:**
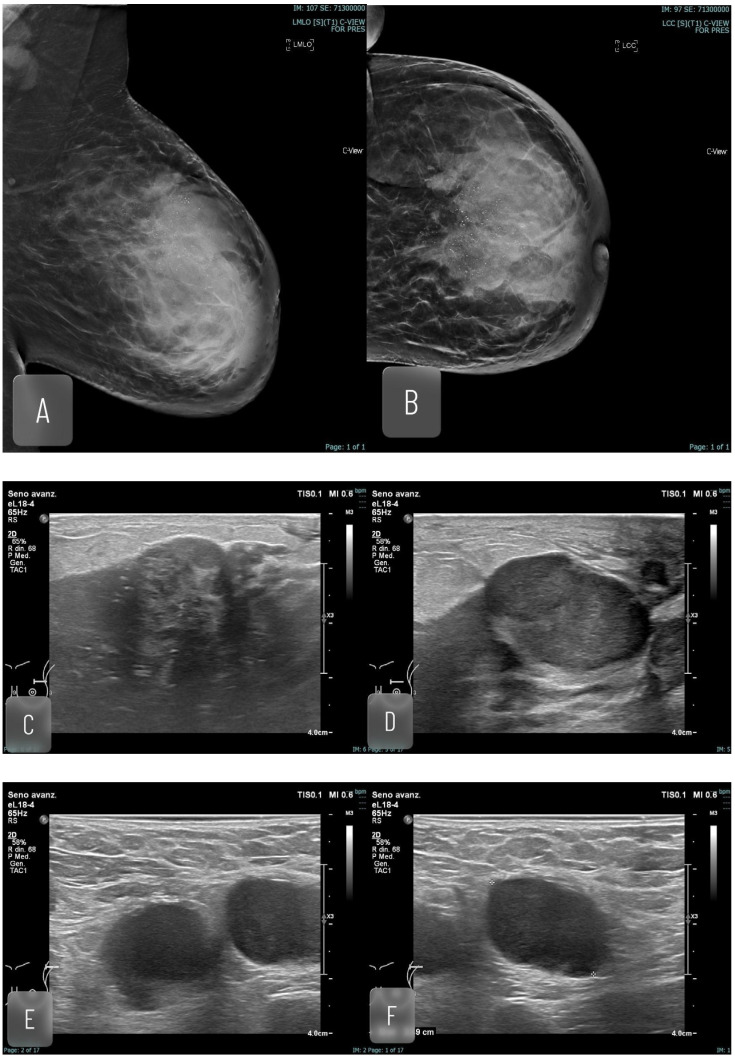

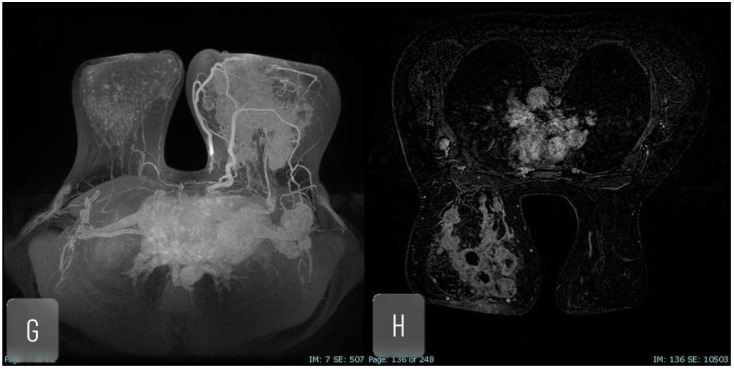
Forty-two yo. breastfeeding woman, with swollen left breast, not responding to antibiotics. Prior colorectal cancer treated with surgery and chemotherapy (Patient with known Lynch Syndrome). Mammography detected a large radiopaque area (70 mm) with calcifications. A core needle biopsy was performed, which detected high grade ductal infiltrant carcinoma associated with DCIS component (high nuclear grade). In homolateral axillary cave lymphadenopathy was detected (cytological examination revealed lymphonodal involvement). A MRI was performed with a dimensional upgrade of the lesion. (**A**) MLO mammographic projection; (**B**) CC mammographic progression; (**C**,**D**) US imaging, breast lesion; (**E**,**F**) US imaging, axillary lymph node; (**G**,**H**) MRI, MIP and contrast enhanced imaging.

These conditions are clinically important because they closely mimic and are often clinically and radiologically indistinguishable from inflammatory breast cancer (Figure 4). Thorough imaging assessment of these cases is essential. Biopsy is usually indicated to establish the correct diagnosis and to rule out breast cancer.

Given that inflammatory breast cancer represents the primary differential diagnosis in this clinical setting, especially in non-purulent mastitis forms and abscesses, careful evaluation is essential to rule out underlying malignancy. In cases with a presentation consistent with breast abscess, a short-term follow-up—typically 7 to 14 days after antibiotic therapy and drainage—is recommended [40].

In patients presenting with breast erythema and swelling without evidence of abscess on ultrasound—particularly older, non-lactating women or those at elevated breast cancer risk—inflammatory breast cancer should be a key diagnostic consideration, and mammography should be performed promptly. In lactating women, mammography is generally deferred until acute mastitis symptoms improve after antibiotic therapy; however, it is warranted in cases of suspected malignancy or with a prolonged or atypical clinical course.

Mastitis and breast abscess may mimic inflammatory breast cancer, but certain imaging characteristics can aid differentiation. Inflammatory breast cancer typically presents with diffuse skin thickening, whereas in mastitis or abscess the thickening is usually localized to the affected area. Suspicious microcalcifications are highly specific for malignancy in patients with inflammatory breast symptoms of uncertain origin. Ultrasound findings of a mass with a hypoechoic wall and interstitial fluid favor abscess over cancer. Axillary node assessment can also help: metastatic nodes in malignancy often show marked enlargement, cortical thickening, and hilar displacement, while reactive lymphadenopathy in abscess is characterized by mild, diffuse cortical thickening. On MRI, inflammatory breast cancer more often demonstrates heterogeneous enhancement with washout kinetics, whereas abscesses show high T2 signal and benign enhancement patterns.

Breast biopsy is essential in patients with persistent symptoms or antibiotic-refractory disease and should be performed promptly [41].

## 6. Imaging Findings in Mastitis and Breast Cancer

### 6.1. Ultrasound (US) and Contrast-Enhanced Ultrasound (CEUS)

Ultrasound is the first-line imaging modality for evaluating suspected mastitis, given its ability to characterize parenchymal and superficial changes. In mastitis, US typically reveals increased skin thickness, diffuse subcutaneous edema, and irregular hypoechoic areas. Abscesses appear as hypoechoic lesions, which may be well-circumscribed or irregular with internal septations. The echographic appearance varies with disease stage and type; granulomatous mastitis often presents as multiple irregular hypoechoic masses with tubular extensions [42]. Breast inflammation may extend to the axillary lymph nodes, causing reactive lymphadenopathy. Benign reactive nodes generally show smooth cortical thickening with preservation of the fatty hilum, although differentiation from malignant lymphadenopathy can be challenging based on imaging alone. In breast carcinoma [30], US typically demonstrates irregular, hypoechoic masses with non-circumscribed or spiculated margins, often accompanied by posterior acoustic shadowing. Tumor-involved lymph nodes may exhibit irregular cortical thickening and partial or complete loss of the fatty hilum. Doppler evaluation can reveal increased intralesional and perilesional vascularity, while elastography frequently shows higher stiffness compared with inflammatory lesions. CEUS improves the evaluation of breast microcirculation and lesion vascularity. Granulomatous mastitis tends to display prolonged time-to-peak (TTP) and lower wash-in slope (WIS) values compared with carcinoma, reflecting different perfusion dynamics. Malignant lesions generally exhibit a shorter TTP and steeper WIS due to their neoangiogenic patterns [30,39].

### 6.2. Mammography

**Figure 5:**
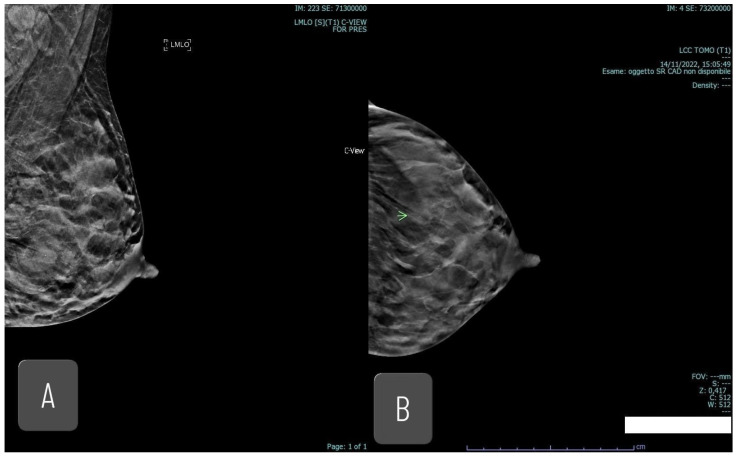

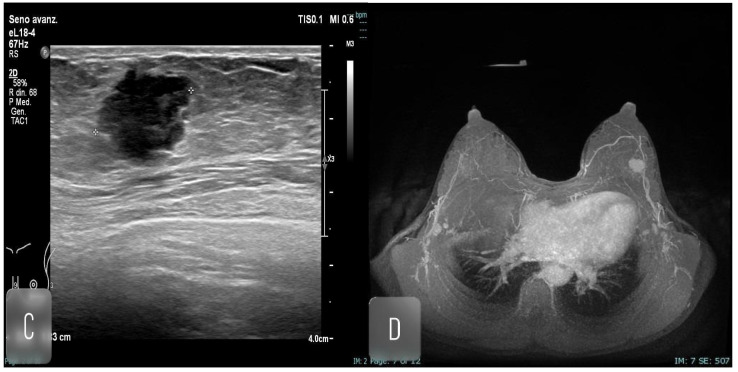
Forty-one yo. breastfeeding woman, with palpable lump in left breast. Mammography detected a radiopacity (20 mm). Core needle biopsy reveals high grade apocrine invasive carcinoma. (**A**,**B**) MLO and CC mammography projections; (**C**) US imaging; (**D**) MRI, MIP.

Mammography is less sensitive for detecting mastitis, particularly in its acute phase, and findings are often non-specific. Possible features include skin thickening, increased density, and architectural distortion; abscesses are frequently not visible. In cases of duct ectasia, mammography may reveal thick, linear calcifications oriented toward the nipple. In breast cancer, mammography is more specific, often revealing masses (Figure 5), suspicious calcifications, or architectural distortion. Malignant calcifications tend to be smaller, irregular, and clustered. Mammography is essential for detecting early cancer signs such as microcalcifications, which are not typical of mastitis.

### 6.3. Magnetic Resonance Imaging (MRI)

MRI in mastitis may demonstrate non-mass enhancement or heterogeneous enhancement patterns, particularly in granulomatous forms. T2-weighted sequences can show dilated ducts containing fat [43]. In breast cancer, MRI offers high sensitivity, especially in dense breasts, providing accurate assessment of tumor extent and evaluation of involvement of adjacent structures [43].

## 7. Treatment and Management

### 7.1. Lactational Mastitis

Effective management of lactational mastitis focuses on symptom relief, reduction in inflammation and addressing underlying causes. Many cases resolve without the need for antibiotics. Supportive care includes rest, adequate hydration, continued on-demand breastfeeding, avoidance of nipple shields, use of a supportive bra, and lymphatic drainage massage. Additionally, patients should use breast pumps only when necessary, ensure a proper fit, and avoid nipple trauma by using moderate suction levels. The use of NSAIDs (non-Steroidal Anti-Inflammatory Drugs) and ice packs can help alleviate pain and swelling [14].

Antibiotic treatment is indicated to bacterial infections confirmed by persistent symptoms that do not improve with supportive care or by positive culture results.

Prophylactic use of antibiotics is not recommended, as it can disrupt the breast microbiome and promote antimicrobial resistance.

The following regimens are recommended:

− First-line treatments: Dicloxacillin, flucloxacillin, or cephalexin for 10 to 14 days, targeting Gram-positive organisms [44].
− Second-line treatments: Clindamycin 300 mg 4 times daily for 10 to 14 days or trimethoprim-sulfamethoxazole double-strength twice daily for 10 to 14 days are alternatives, particularly for resistant infections if not contraindicated (e.g., patients with G6PD deficiency) [45].

Hospitalization is reserved for severe cases requiring intravenous antibiotics, with rooming-in encouraged to support continued breastfeeding. In hospitalized patients, empiric treatment with vancomycin should be initiated until culture and sensitivity results are available. 

### 7.2. Periductal Mastitis

Although rare, PDM is a painful condition often resistant to conservative treatments. Surgery is the most common intervention, with various techniques tailored to optimize outcomes, minimize recurrence, and ensure cosmetic acceptability. In addition to surgical management, acute PDM should be treated with broad-spectrum antibiotics to control inflammation; however, antibiotic therapy alone was shown to have poor outcomes. Treatment should be individualized, taking into account disease severity, patient preferences, and the surgeon’s expertise.

Breast duct irrigation and minor excision techniques are preferred for less severe cases, while extensive excision or plastic surgery is more suitable for advanced or recurrent PDM. Identifying the optimal approach remains challenging due to variability in reported outcomes across studies. Minimally invasive procedures—including incision and drainage, simple incision, and breast duct irrigation—have demonstrated mixed results.

### 7.3. Idiopathic Granulomatous Mastitis

Management of IGM encompasses various approaches: observation, antibiotics, corticosteroids, immunosuppressants, and surgery. Corticosteroid therapy is commonly employed, often in combination with other modalities [46].

Although IGM can be self-limiting, its prolonged course and potential impact on quality of life warrant individualized treatment plans.

An interprofessional approach, tailored to disease severity and patient preferences, is recommended to achieve symptomatic control while minimizing adverse effects. 

Generally, IGM has a self-limited course and can resolve without treatment in approximately two years, with spontaneous improvement in as little as 6–12 months [47]. In IGM, medical treatment is preferred to avoid surgery. Indeed, surgical treatment often leads to poor cosmetic outcomes and is not recommended due to poor wound healing and risk of recurrence. 

Medical treatment includes observation, antibiotics, steroids (both intralesional injection or oral subministration), and immune modulators such as methotrexate [47]. 

While the role of antibiotics in IGM remains debated, they are sometimes used, particularly when bacterial infections like Corynebacterium are suspected (e.g., when an abscess is present, due to high prevalence of Corynebacterium infection in CNGM—cystic neutrophilic granulomatous mastitis). 

When a diagnosis is established, steroid therapy is warranted to warrant symptomatic control and to control inflammation [47]. 

For cases unresponsive to corticosteroids, methotrexate has shown efficacy, with remission rates as high as 93%. Its use requires careful monitoring for side effects like myelosuppression [46]. 

### 7.4. Patient Education 

In general, promoting awareness of symptoms, encouraging smoking cessation, maintaining proper breast hygiene, and ensuring regular follow-up for high-risk individuals are essential measures for the effective prevention and management of acute mastitis. For lactational mastitis, patients should be encouraged to practice regular and complete breast emptying during breastfeeding, alternate feeding positions to ensure adequate drainage, and address nipple damage promptly to prevent infection. Proper hygiene and early recognition of symptoms—such as localized pain, redness, and swelling—are crucial for timely treatment. Prevention of periductal mastitis (PDM) focuses on smoking cessation, as it significantly reduces the risk of recurrence, and consideration of breast duct resection in high-risk patients. Patients should be informed about the recurrent nature of PDM and the importance of consistent follow-up care. For idiopathic granulomatous mastitis (IGM), although the exact cause remains unclear, early detection and appropriate management—including antibiotics for potential *Corynebacterium* infections—can help prevent complications. Patients should be educated about the possibility of spontaneous remission and the need for individualized treatment plans, which may involve corticosteroids, surgery, or antibiotics, with careful consideration of cosmetic outcomes following surgery [48].

## 8. Conclusions and Future Perspectives

Radiological imaging remains essential for evaluating mastitis, enabling detailed assessment of parenchymal and superficial changes, guiding interventional procedures, and aiding in the distinction between benign inflammatory and malignant conditions. However, imaging findings are often non-specific and can substantially overlap between mastitis subtypes and breast cancer, necessitating careful correlation with clinical history, physical examination, and, when indicated, histopathological confirmation [39].

Although no imaging technologies have been exclusively developed for mastitis diagnosis, advances in breast imaging modalities—particularly high-resolution ultrasound, contrast-enhanced ultrasound (CEUS), digital breast tomosynthesis (DBT), and MRI—are improving diagnostic confidence. CEUS, through quantitative parameters such as time-to-peak (TTP), wash-in slope (WIS), and MRI, combining dynamic contrast-enhanced (DCE) and diffusion-weighted imaging (DWI) analysis, have shown promise in differentiating inflammatory from malignant lesions [49].

Beyond inflammatory breast cancer, mastitis can be part of the differential diagnosis with other breast malignancies, particularly in clinically atypical or persistent cases [6]. Ductal carcinoma in situ (DCIS) with extensive peritumoral edema can resemble an inflammatory process, while some aggressive invasive ductal carcinomas—especially triple-negative and medullary subtypes—may present with marked hyperemia and skin thickening. Invasive lobular carcinoma, due to its tendency to cause diffuse thickening and skin retraction without a discrete mass, can also be mistaken for chronic or sclerosing mastitis. Therefore, in mastitis with an atypical course, it is crucial to maintain a broad oncologic differential and perform timely biopsy.

Artificial intelligence (AI) and machine learning (ML) are set to further bolster diagnostic capabilities. In a study comparing deep learning-based AI to sonographer interpretations in non-lactating mastitis versus malignant tumors, the AI system demonstrated an accuracy of 85.3%, sensitivity of 83.0%, and specificity of 87.2% [49]. Radiomics with ultrasound and deep learning—such as a DLRN nomogram—achieved AUC = 0.84 in differentiating mass mastitis from invasive breast cancer. AI-enabled semi-automated segmentation has also shown feasibility in extracting ultrasound radiomic features from focal breast lesions, though further validation is needed [49,50].

Radiologist expertise remains a decisive factor for diagnostic accuracy. Familiarity with the spectrum of presentations for different mastitis subtypes—lactational, periductal, granulomatous, infectious—and potential complications is essential to avoid misinterpretation and unnecessary interventions. Persistent or atypical mastitis, particularly when refractory to adequate antibiotic therapy, should always prompt biopsy to exclude malignancy.

Future directions should include large-scale prospective studies to validate imaging biomarkers, AI-enhanced workflows, and multimodal diagnostic algorithms in mastitis. Standardizing imaging protocols and diagnostic criteria may reduce inter-observer variability and improve outcomes. Preventive strategies—such as patient education, smoking cessation, and prompt symptom recognition—should remain integral parts of clinical management, alongside personalized therapy plans aimed at reducing recurrence and preserving cosmetic integrity.

## Figures and Tables

**Table 1:** Different types of mastitis synthesis: diagnostic tools and imaging features.

Type	Subtype	Age of Onset	Mammography	Ultrasound	Magnetic Resonance
Lactational Mastitis		Reproductive age	Non-specific findings	Skin thickening, increased echogenicity of subcutaneous fat, hypoechoic fluid area, peripheral hyperemia.	Intense BPE, edema, and non-mass enhanced pattern. If abscess is present: well-defined lesion with peripheral rim enhancement and a hyperintense T2-weighted center.
Non lactational mastitis	Periductal mastitis	Non lactating women	Non-specific findings	Hypoechoic mass Internal tubular pattern.	Non-mass enhancement, rim-enhancing if abscesses are present, evidence of fistula.
	Idiopathic Granulomatous Mastitis	Reproductive age	Non-specific findings	Hypoechoic mass, tubular extensions, reticular pattern. Lesions typically appear parallel to the skin.	Segmental heterogeneous enhancement, ring-like abscess. If mass-like lesions is present: round shape, smooth margins, rim enhancement, peripheral rim enhancement.
	Inflammatory breast carcinoma	Generally adult age	Suspicious microcalcification, diffuse skin thickening	Diffuse skin thickening, hypoechoic lesion. Metastatic nodes with marked enlargement, cortical thickening, and hilar displacement.	Heterogeneous enhanced lesion with washout kinetics.

## Data Availability

No new data were created or analyzed in this study. Data sharing is not applicable to this article.

## Abbreviations

LM	lactational mastitis
PM	puerperal mastitis
NLM	non-lactational mastitis
IGM	idiopathic granulomatous mastitis
MRI	magnetic resonance imaging
PDM	periductal mastitis
DMP	diabetic mastopathy
BC	breast cancer
LM	lupus mastitis
TM	tubercular mastitis
US	ultrasound
CEUS	contrast enhanced ultrasound
CEM	contrast enhanced mammography
BPE	background parenchymal enhancement
NME	non-mass enhancement
TTP	time to peak
DBT	digital breast tomosynthesis

## References

[1] Kaplan R.L. (2024). Neonatal Mastitis: Clinical Presentation and Approach to Evaluation and Management. Pediatr. Emerg. Care.

[2] Costa Morais Oliveira V., Cubas-Vega N., López Del-Tejo P., Baía-da-Silva D.C., Araújo Tavares M., Picinin Safe I., Cordeiro-Santos M., Lacerda M.V.G., Val F. (2021). Non-lactational Infectious Mastitis in the Americas: A Systematic Review. Front. Med..

[3] Mitchell K.B., Johnson H.M., Rodrıguez J.M., Eglash A., Scherzinger C., Zakarija-Grkovic I., Widmer Cash K., Berens P., Miller B., the Academy of Breastfeeding Medicine (2022). Academy of Breastfeeding Medicine Clinical Protocol #36: The Mastitis Spectrum, Revised 2022. Breastfeed. Med..

[4] Wilson E., Woodd S.L., Benova L. (2020). Incidence of and Risk Factors for Lactational Mastitis: A Systematic Review. J. Hum. Lact..

[5] Singla D.V., Garg D.D., Dua D.A., Bal D.A., Singh D.T., Prabhakar D.N., Dahiya D.D. (2025). Imaging enigma in mastitis: A comprehensive study of multifaceted causes, clinical and radiological presentations. Curr. Probl. Diagn. Radiol..

[6] Poyraz N., Emlik G.D., Batur A., Gundes E., Keskin S. (2016). Magnetic Resonance Imaging Features of Idiopathic Granulomatous Mastitis: A Retrospective Analysis. Iran. J. Radiol..

[7] Wolfrum A., Kümmel S., Theuerkauf I., Pelz E., Reinisch M. (2018). Granulomatous Mastitis: A Therapeutic and Diagnostic Challenge. Breast Care.

[8] Dilaveri C., Degnim A., Lee C., DeSimone D., Moldoveanu D., Ghosh K. (2024). Idiopathic Granulomatous Mastitis. Breast J..

[9] Jiao Y., Chang K., Jiang Y., Zhang J. (2023). Identification of periductal mastitis and granulomatous lobular mastitis: A literature review. Ann. Transl. Med..

[10] Khanna R., Prasanna G.V., Gupta P., Kumar M., Khanna S., Khanna A.K. (2002). Mammary tuberculosis: Report on 52 cases. Postgrad. Med. J..

[11] Kataria K., Srivastava A., Dhar A. (2013). Management of lactational mastitis and breast abscesses: Review of current knowledge and practice. Indian. J. Surg..

[12] Peterson M.S., Gegios A.R., Elezaby M.A., Salkowski L.R., Woods R.W., Narayan A.K., Strigel R.M., Roy M., Fowler A.M. (2023). Breast Imaging and Intervention during Pregnancy and Lactation. Radiographics.

[13] Douglas P. (2022). Re-thinking benign inflammation of the lactating breast: Classification, prevention, and management. Womens Health.

[14] Boakes E., Woods A., Johnson N., Kadoglou N. (2018). Breast Infection: A Review of Diagnosis and Management Practices. Eur. J. Breast Health.

[15] Zhou F., Liu L., Wang F., Yu L., Xiang Y., Zheng C., Huang S., Yang Z., Yu Z. (2024). Periductal Mastitis, a Disease with Distinct Clinicopathological Features from Granulomatous Lobular Mastitis. J. Inflamm. Res..

[16] Liu L., Zhou F., Wang P., Yu L., Ma Z., Li Y., Gao D., Zhang Q., Li L., Yu Z. (2017). Periductal Mastitis: An Inflammatory Disease Related to Bacterial Infection and Consequent Immune Responses?. Mediat. Inflamm..

[17] Xu H., Liu R., Lv Y., Fan Z., Mu W., Yang Q., Fu H., Li Y. (2022). Treatments for Periductal Mastitis: Systematic Review and Meta-Analysis. Breast Care.

[18] Wong S.C.Y., Poon R.W.S., Chen J.H.K., Tse H., Lo J.Y.C., Ng T.K., Au J.C.K., Tse C.W.S., Cheung I.Y.Y., Yuk M.T. (2017). *Corynebacterium kroppenstedtii* Is an Emerging Cause of Mastitis Especially in Patients With Psychiatric Illness on Antipsychotic Medication. Open Forum Infect. Dis..

[19] Krawczyk N., Kühn T., Ditsch N., Hartmann S., Gentilini O.D., Lebeau A., de Boniface J., Hahn M., Çakmak G.K., Alipour S. (2024). Idiopathic Granulomatous Mastitis as a Benign Condition Mimicking Inflammatory Breast Cancer: Current Status, Knowledge Gaps and Rationale for the GRAMAREG Study (EUBREAST-15). Cancers.

[20] Kasales C.J., Han B., Smith J.S., Chetlen A.L., Kaneda H.J., Shereef S. (2014). Nonpuerperal mastitis and subareolar abscess of the breast. AJR Am. J. Roentgenol..

[21] Mariano L., Nicosia L., Scolari S., Pasi S., Netti S., Mazzarol G., Latronico A., Cassano E. (2024). Diabetic Mastopathy: A Monocentric Study to Explore This Uncommon Breast Disease. Diagnostics.

[22] Wilsher M.J., Banerjee D. (2021). Eosinophilic mastitis in a lactating breast. Pathology.

[23] Haydar M., Maamar Y., Al Shabab M., Dawli W., Roumieh D., Al-Shehabi Z. (2024). Isolated eosinophilic mastitis mimicking carcinoma: A rare case report from Syria. Int. J. Surg. Case Rep..

[24] Nagy S., Daniel K., Kesselman M.M. (2025). Imaging Findings of Lupus Mastitis: A Systematic Review of Case Studies. Cureus.

[25] Chen X., Hoda S.A., Delellis R.A., Seshan S.V. (2005). Lupus mastitis. Breast J..

[26] Erivwo P., Turashvili G. (2021). Pathology of IgG4-related sclerosing mastitis. J. Clin. Pathol..

[27] Pluguez-Turull C.W., Nanyes J.E., Quintero C.J., Alizai H., Mais D.D., Kist K.A., Dornbluth N.C. (2018). Idiopathic Granulomatous Mastitis: Manifestations at Multimodality Imaging and Pitfalls. Radiographics.

[28] Catalano O., Fusco R., De Muzio F., Simonetti I., Palumbo P., Bruno F., Borgheresi A., Agostini A., Gabelloni M., Varelli C. (2023). Recent Advances in Ultrasound Breast Imaging: From Industry to Clinical Practice. Diagnostics.

[29] Zheng Y., Wang L., Han X., Shen L., Ling C., Qian Z., Zhu L., Dong F., Han Q. (2022). Combining contrast-enhanced ultrasound and blood cell analysis to improve diagnostic accuracy of plasma cell mastitis. Exp. Biol. Med..

[30] Yin L., Agyekum E.A., Zhang Q., Pan L., Wu T., Xiao X., Qian X.Q. (2022). Differentiation Between Granulomatous Lobular Mastitis and Breast Cancer Using Quantitative Parameters on Contrast-Enhanced Ultrasound. Front. Oncol..

[31] Shanbhag N.M., Ameri M.A., Shanbhag S.N., Anandan N., Balaraj K., Bin Sumaida A. (2024). Diagnostic Challenges and Insights Into Granulomatous Mastitis: A Systematic Review. Cureus.

[32] Mohindra N., Jain N., Sabaretnam M., Agrawal V., Mishra P., Chaturvedi P., Mishra A., Agarwal G. (2023). Mammography and Digital Breast Tomosynthesis in Granulomatous and Nongranulomatous Mastitis. J. Surg. Res..

[33] Jochelson M.S., Lobbes M.B.I. (2021). Contrast-enhanced Mammography: State of the Art. Radiology.

[34] Lorente-Ramos R.M., Azpeitia-Armán J., Oliva-Fonte C., Pérez-Bartolomé A., Azpeitia Hernández J. (2023). Contrast-enhanced Mammography Artifacts and Pitfalls: Tips and Tricks to Avoid Misinterpretation. Radiographics.

[35] Xu K., Chung M., Hayward J.H., Kelil T., Lee A.Y., Ray K.M. (2024). MRI of the Lactating Breast. Radiographics.

[36] Langer A.K. (2020). Breast Imaging in Pregnancy and Lactation. Adv. Exp. Med. Biol..

[37] Zhang L., Hu J., Guys N., Meng J., Chu J., Zhang W., Liu A., Wang S., Song Q. (2018). Diffusion-weighted imaging in relation to morphology on dynamic contrast enhancement MRI: The diagnostic value of characterizing non-puerperal mastitis. Eur. Radiol..

[38] Cheng L., Reddy V., Solmos G., Watkins L., Cimbaluk D., Bitterman P., Ghai R., Gattuso P. (2015). Mastitis, a Radiographic, Clinical, and Histopathologic Review. Breast J..

[39] Chang C.M., Lin M.C., Yin W.Y. (2019). Risk of breast cancer in women with non-lactational mastitis. Sci. Rep..

[40] Leong P.W., Chotai N.C., Kulkarni S. (2018). Imaging Features of Inflammatory Breast Disorders: A Pictorial Essay. Korean J. Radiol..

[41] Guirguis M.S., Adrada B., Santiago L., Candelaria R., Arribas E. (2021). Mimickers of breast malignancy: Imaging findings, pathologic concordance and clinical management. Insights Imaging.

[42] Febery A., Bennett I. (2019). Sonographic features of inflammatory conditions of the breast. Australas. J. Ultrasound Med..

[43] Soylu Boy F.N., Esen Icten G., Kayadibi Y., Tasdelen I., Alver D. (2023). Idiopathic Granulomatous Mastitis or Breast Cancer? A Comparative MRI Study in Patients Presenting with Non-Mass Enhancement. Diagnostics.

[44] Spencer J.P. (2008). Management of mastitis in breastfeeding women. Am. Fam. Physician.

[45] Pustotina O. (2016). Management of mastitis and breast engorgement in breastfeeding women. J. Matern. Fetal Neonatal Med..

[46] Akbulut S., Arikanoglu Z., Senol A., Sogutcu N., Basbug M., Yeniaras E., Yagmur Y. (2011). Is methotrexate an acceptable treatment in the management of idiopathic granulomatous mastitis?. Arch. Gynecol. Obs..

[47] Benson J.R., Dumitru D. (2016). Idiopathic granulomatous mastitis: Presentation, investigation and management. Future Oncol..

[48] Stary C.M., Lee Y.S., Balfour J. (2011). Idiopathic granulomatous mastitis associated with *corynebacterium* sp. *Infection*. Hawaii. Med. J..

[49] Zhou Y., Feng B.J., Yue W.W., Liu Y., Xu Z.F., Xing W., Xu Z., Yao J.C., Wang S.R., Xu D. (2022). Differentiating non-lactating mastitis and malignant breast tumors by deep-learning based AI automatic classification system: A preliminary study. Front. Oncol..

[50] Wu L., Li S., Wu C., Wu S., Lin Y., Wei D. (2024). Ultrasound-based deep learning radiomics nomogram for differentiating mass mastitis from invasive breast cancer. BMC Med. Imaging.

